# Temperature profile and residual heat of monopolar laparoscopic and endoscopic dissection instruments

**DOI:** 10.1007/s00464-021-08804-4

**Published:** 2021-10-27

**Authors:** Franz Brinkmann, Ronny Hüttner, Philipp J. Mehner, Konrad Henkel, Georgi Paschew, Moritz Herzog, Nora Martens, Andreas Richter, Sebastian Hinz, Justus Groß, Clemens Schafmayer, Jochen Hampe, Alexander Hendricks, Frank Schwandner

**Affiliations:** 1 Department of Medicine I, University Hospital Dresden, Technische Universität Dresden (TU Dresden), Dresden, Germany; 2 Chair of Microsystems, Faculty of Electrical and Computer Engineering, Technische Universität Dresden (TU Dresden), Dresden, Germany; 3 Else Kröner Fresenius Center for Digital Health, Technische Universität Dresden (TU Dresden), Dresden, Germany; 4 Department of General, Visceral, Vascular and Transplantation Surgery, University Medical Center Rostock, Rostock, Germany; 5 Department of Medicine I, University Hospital Carl Gustav Carus, Technische Universität Dresden (TU Dresden), Fetscherstr. 74, 01307 Dresden, Germany

**Keywords:** Residual heat, Electrosurgical device, Endoscopic submucosal dissection, Temperature profile

## Abstract

**Background:**

Endoscopic and laparoscopic electrosurgical devices (ED) are of great importance in modern medicine but can cause adverse events such as tissue injuries and burns from residual heat. While laparoscopic tools are well investigated, detailed insights about the temperature profile of endoscopic knives are lacking. Our aim is to investigate the temperature and the residual heat of laparoscopic and endoscopic monopolar instruments to increase the safety in handling ED.

**Methods:**

An infrared camera was used to measure the temperature of laparoscopic and endoscopic instruments during energy application and to determine the cooling time to below 50 °C at a porcine stomach. Different power levels and cutting intervals were studied to investigate their impact on the temperature profile.

**Results:**

During activation, the laparoscopic hook exceeded 120 °C regularly for an up to 10 mm shaft length. With regards to endoknives, only the Dual Tip Knife showed a shaft temperature of above 50 °C. The residual heat of the laparoscopic hook remained above 50 °C for at least 15 s after activation. Endoknives cooled to below 50 °C in 4 s. A higher power level and longer cutting duration significantly increased the shaft temperature and prolonged the cooling time (*p* < 0.001).

**Conclusion:**

Residual heat and maximum temperature during energy application depend strongly on the chosen effect and cutting duration. To avoid potential injuries, the user should not touch any tissue with the laparoscopic hook for at least 15 s and with the endoknives for at least 4 s after energy application. As the shaft also heats up to over 120 °C, the user should be careful to avoid tissue contact during activation with the shaft. These results should be strongly considered for safety reasons when handling monopolar ED.

In more than 80% of today’s performed surgical procedures electrosurgical devices (ED) are used [1]. This practice has a long history reaching back to the Egyptians who used cautery already in 3000 BC to treat hemorrhagic shocks [2]. The first modern electrosurgical instruments and devices were invented in the 1920ies by Christian Erbe followed shortly thereafter by the first commercially available electrosurgical unit by Bovie [3, 4]. With the rise of minimal invasive procedures, electrosurgery became more and more important until today.

## Safety aspects of electrosurgery in laparoscopy and endoscopy

Despite significant benefits in tissue dissection and hemostasis, serious adverse events can occur with electrosurgery. Several studies indicate that more than 50% of medical adverse events are related to surgical procedures [5] and 15.9% of incidents during surgical procedures are equipment associated [6]. In particular, electrosurgery is related with risks that may seriously impair the outcome [7], such as thermal injury related to residual heat when the hot instrument accidently touches further tissue creating the risk of injuries. Additional laparoscopic energy related complications are insulation failure, capacitive coupling, direct coupling, and direct application [8]. Apart from the common laparoscopic complications that arise due to surgical error, another major complication from electrosurgery are thermal injuries to adjacent organs [9]. Site burns (e.g. pads, prostheses, surgeon hand) occur more frequently while using electrosurgery [10]. There are 40,000 cases of patient burns from electrosurgical equipment each year in the USA [8]. The prevalence of bowel injuries related to electrosurgery is estimated at 1–2 per 1000 patients with a high morbidity related to unrecognized injuries [11]. Many of these adverse events are preventable by ensuring a profound understanding of the technology and an awareness of potential risks [12]. Most complications are based on the incorrect use or lack of understanding of the instruments and settings. Therefore, knowledge in operating electrosurgical devices is of great importance, but surgeons regularly use energy-based devices whose principles and functions are not fully understood. To address this problem, the Society of American Gastrointestinal and Endoscopic Surgeons has initiated the Fundamental Use of Surgical Energy program to develop an educational curriculum. Latest studies found many gaps in the knowledge about the safe use of electrosurgical devices [13, 14].

Electrosurgery is not only used in laparoscopy but also in many endoscopic therapeutic procedures with a similar spectrum of adverse events. For example the electrocoagulation syndrome, defined as a transmural burn of the colon wall, occurs with an incidence of 8.6–9.5% [15, 16]. With the rise of the endoscopic submucosal dissection (ESD) as the standard procedure for early gastrointestinal neoplasia’s, deductively, monopolar endoscopic dissection knives (endoknives) were used more frequently in endoscopy. The most severe complication related to endoscopic electrosurgery is thermal injury of the tissue leading to the perforation of the bowel wall (in 4% for esophageal ESD and 4.9% for Colorectal ESD) [15, 17, 18].

## Temperature profile of electrosurgical instruments

Recent studies which investigated the peak temperature of laparoscopic instruments and the thermal spread during activation, demonstrated the widespread of the heat detected by real time infrared thermography. They exhibited, that the temperature of the tip of the laparoscopic energy devices is still increased to over 100 °C for multiple seconds after usage and could therefore cause serious tissue damage [19, 20]. As denaturation of intracellular proteins and destruction of cell membranes occur when the cells are heated to above 45–50 °C, an adequate cooling period after usage is crucial [21, 22]. Govekar et al. stated that the residual heat is overlooked and should be taken into further consideration [23, 24]. Preliminary studies showed that an increased power level and longer cutting time are also associated with increased temperature [25]. However, the temperature of the whole instruments including the shaft were not analyzed [26]. Furthermore, the tissue is not only touched with the tip of the instrument but -often accidentally- also with parts of the shaft. To prevent adverse events, surgeons should be well informed about the peak heat and residual heat of the entire endoscopic and laparoscopic dissection knives, which also includes the shaft of the instrument. To design optimized and innovative endoscopes and laparoscopic tools, the temperature profiles of these instruments need to be known and taken account of during the procedure. Besides the analysis of the temperature profile of laparoscopic instruments we aimed to investigate endoknives, too, as detailed studies in this area are still lacking.

## Methods

**Fig. 1:**
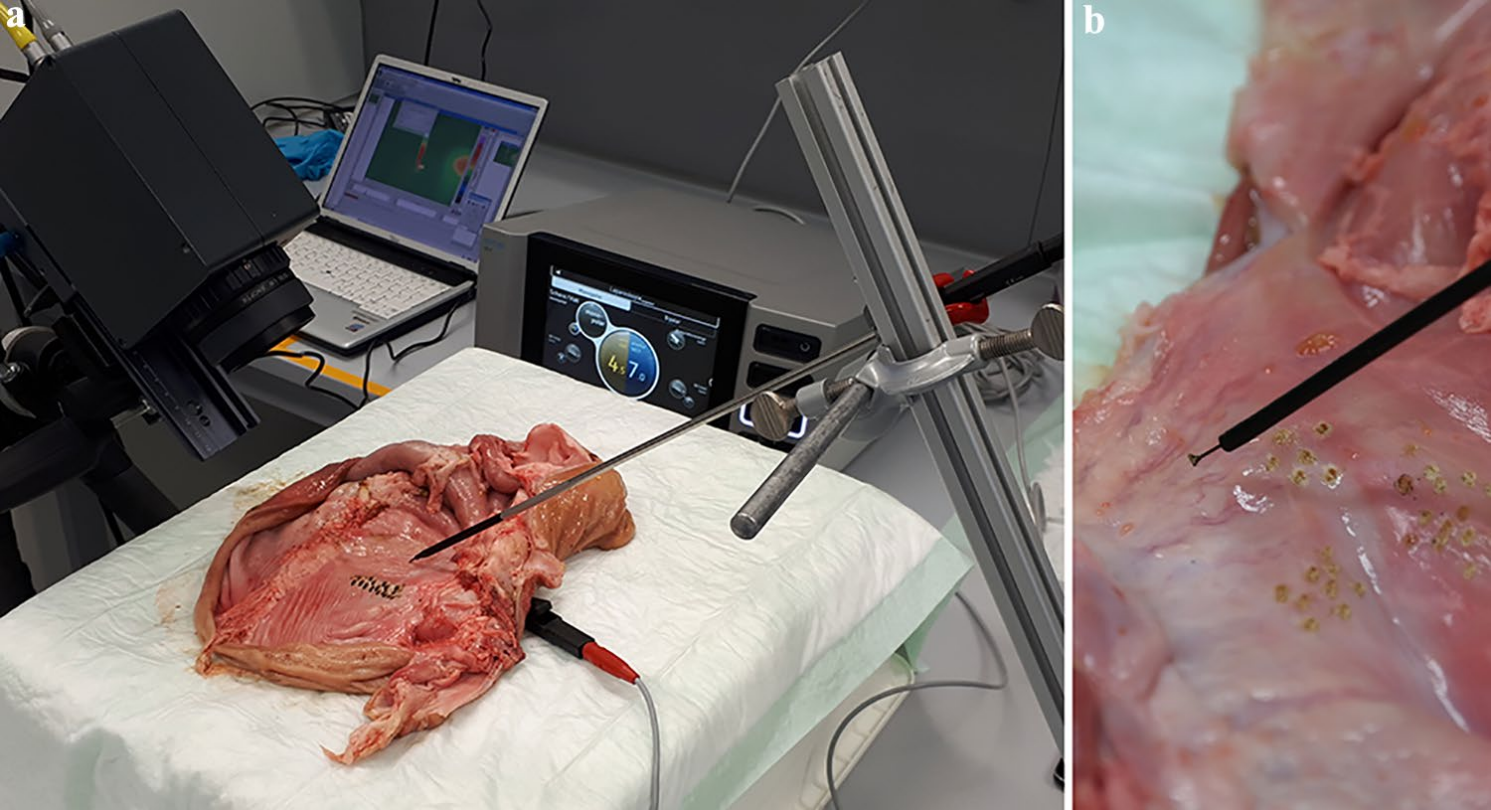
Experiment setup. **a** Experiment setup including VIO® 3 electrosurgery unit by Erbe®, an infrared camera, a porcine stomach, and a laparoscopic hook. **b** Experiment setup with a porcine stomach and the endoscopic Triangle Tip Knife

Electrosurgery was performed using the VIO® *3* electrosurgery unit (No: 10160-000) (Fig. 1a) with a maximum power output of 400 Watt. In this study, we used the L-Hook electrode (Erbe®, Item No. 20191-161, Diameter 5 mm, shaft insulated, length 320 mm), a standard monopolar laparoscopic instrument in laparoscopic dissections (laparoscopic hook). With regards to endoscopy, we tested the single-use monopolar electrosurgical Dual Tip Knife (Olympus®, Model KD-650L, diameter: 2.7 mm, working length: 165 cm, cutting knife length: 2.0 mm), the Triangle Tip Knife (Olympus®, Model KD-640L, diameter: 2.7 mm, working length: 165 cm, cutting knife length: 4.5 mm) (Fig. 1b) and the Hook Knife (Olympus®, Model KD-620L, diameter: 2.6 mm, working length: 165 cm, cutting knife length: 4.5 mm). Thermography was performed with an infrared camera (VarioCam® Hr Head 640, Infratec, Germany), which is highly sensitive to radiated wavelengths between 7.5 and 14 µm, using the InfraTec software Irbis for recording (Spatial resolution: 25 × 25 µm). The instruments were painted with a flat black coat (emissivity > 0.9). To collect more detailed temperature data in the biologically relevant range from 30 to 100 °C, we set a cut off at 255 °C as maximum temperature for the infrared camera. A porcine stomach model at room temperature was used to perform the experiments. The experiment setup can be found in Fig. 1a.

To simulate a wide spectrum of realistic operative applications, each instrument was activated in defined intervals for 30 s with a standardized cooling period afterwards. We tested each instrument for the following sequences: A: 1 s activation/2 s pause (10 cycles), B: 3 s activation/2 s pause (6 cycles), C: 5 s cutting/5 s pause (3 cycles), and D: 10 s cutting/5 s pause (2 cycles). After the 30 s activation interval we continued the measurement of the temperature for 60 s to analyze the residual heat. The temperature was measured with 10 fps to record an accurate cooling-period. Due to the not exactly reproducible penetration depth of the marginally different contact angles and tissue adhering to the instrument and changing resistance of the tissue, high standard deviations of the measured temperatures are expected. Each series was repeated three times to minimize these measurement errors. We chose common settings for the electrosurgical cutting mode and power level for clinical comparability. The electrosurgical preciseSect mode by Erbe® is a monopolar cutting mode with coagulation and precise cutting that is regularly used for laparoscopic dissections. In clinical practice, the power level effect 5 is mostly used and was therefore selected in our experiment for the laparoscopic hook. We also included the higher power levels Effect 7 and 10 of the preciseSect mode for comparison. Regarding the endoknives, we performed experiments with the EndoCut Q mode by Erbe®, which is a monopolar and fractionated cutting mode and features alternating cutting and coagulation cycles. Effect 3 is commonly used for endoscopic dissections and was therefore tested. For comparison reasons, we also performed the experiments in the higher power level of effect 4. In the device settings, we selected setting 1 for cut-duration and setting 6 for cut-interval. In accordance with comparable studies [21, 22, 27], we defined an instrument temperature under 50 °C as safe for touching tissue and the time to cool down to below 50 °C as cooling time needed for a safe usage (“time to safety”).

Within our experiment setup, the instruments were mounted at an angle of 45° with the tip contacting the porcine tissue (Fig. 1a). For activation of the electrosurgery the porcine model was slowly moved to cut intact and immaculate tissue. The camera and the instrument were not moved to keep the instrument at a stable angle. The infrared camera was likewise arranged at 45° to avoid distortion effects of the infrared image. During the endoscopic dissection, we injected Gelafusal® solution into the submucosal tissue to simulate realistic operation conditions.

The results were saved as an ASCII file with temperature data for each pixel and frame. Data analysis was performed using Matlab® (Version 2020b). For analysis of the instrument temperature the beginning of the shaft (0 mm shaft length) was defined as the point where the electrode starts to thicken as marked in Fig. 3. The distance to the beginning of the shaft (5 mm and 10 mm shaft length) was calculated using the known instrument diameter to determine the pixel/mm ratio and thus, estimate the distance in millimeters. Data cleaning and statistical analysis was performed in R (RStudio Team (2020), Version 1.3.1093) using the Mann–Whitney-*U*-test/Wilcoxon-test for two groups and two-way analysis of variance (ANOVA) for comparing means of more than two groups. Institutional Review Board (IRB) approval or written consent was not required because no humans/patients or living animals were included in this study.

## Results

**Fig. 2:**
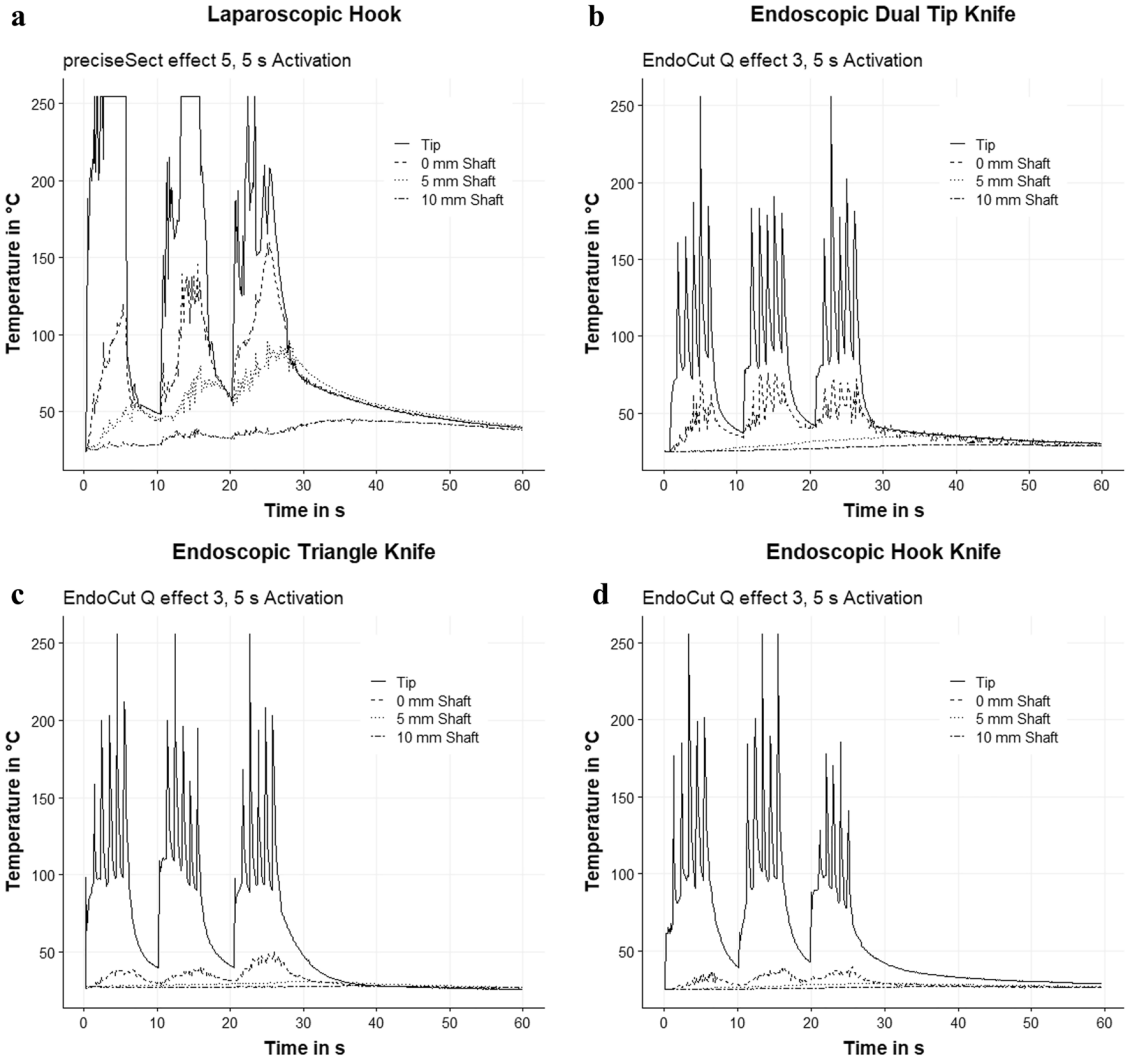
Temperature curve during activation and cooling. **a** Laparoscopic hook. **b** Endoscopic Dual Tip Knife. **c** Endoscopic Hook Knife. **d** Endoscopic Triangle Knife

We generated a vast amount of data for an in-depth analysis of the temperature profile of the investigated instruments. Temperature data were recorded over the entire series. First, we analyzed an exemplary temperature curve for each instrument to receive an overview of the heating and cooling behavior at different measuring points of the instruments (Fig. 2a–d). The laparoscopic hook (Fig. 2a) continues to register an elevated temperature of ≥ 50 °C for longer than 15 s after the end of energy application. In the thermogram, even after 10 s of cooling, significantly elevated temperatures of ≥ 70 °C at the tip and at up to 5 mm shaft length were shown for the laparoscopic hook. After 20 s, temperatures < 50 °C were observed for the laparoscopic hook. The highest temperature during activation was found in the area around the tip. The shaft of the laparoscopic hook also heats up significantly to over 120 °C for up to 5 mm of the shaft length during energy application and needed at least 15 s to cool down to < 50 °C.

The temperature of endoknives (Fig. 2b–d) quickly declines to the initial level after activation. They cooled down to their initial temperature after 5 s of cooling. Regarding the shaft temperature during activation, only the Dual Tip Knife demonstrated an increased temperature of over 50 °C at the shaft, whereas the shaft temperature of the Triangle Knife and the endoscopic Hook Knife did not show any increased temperatures.

**Fig. 3:**
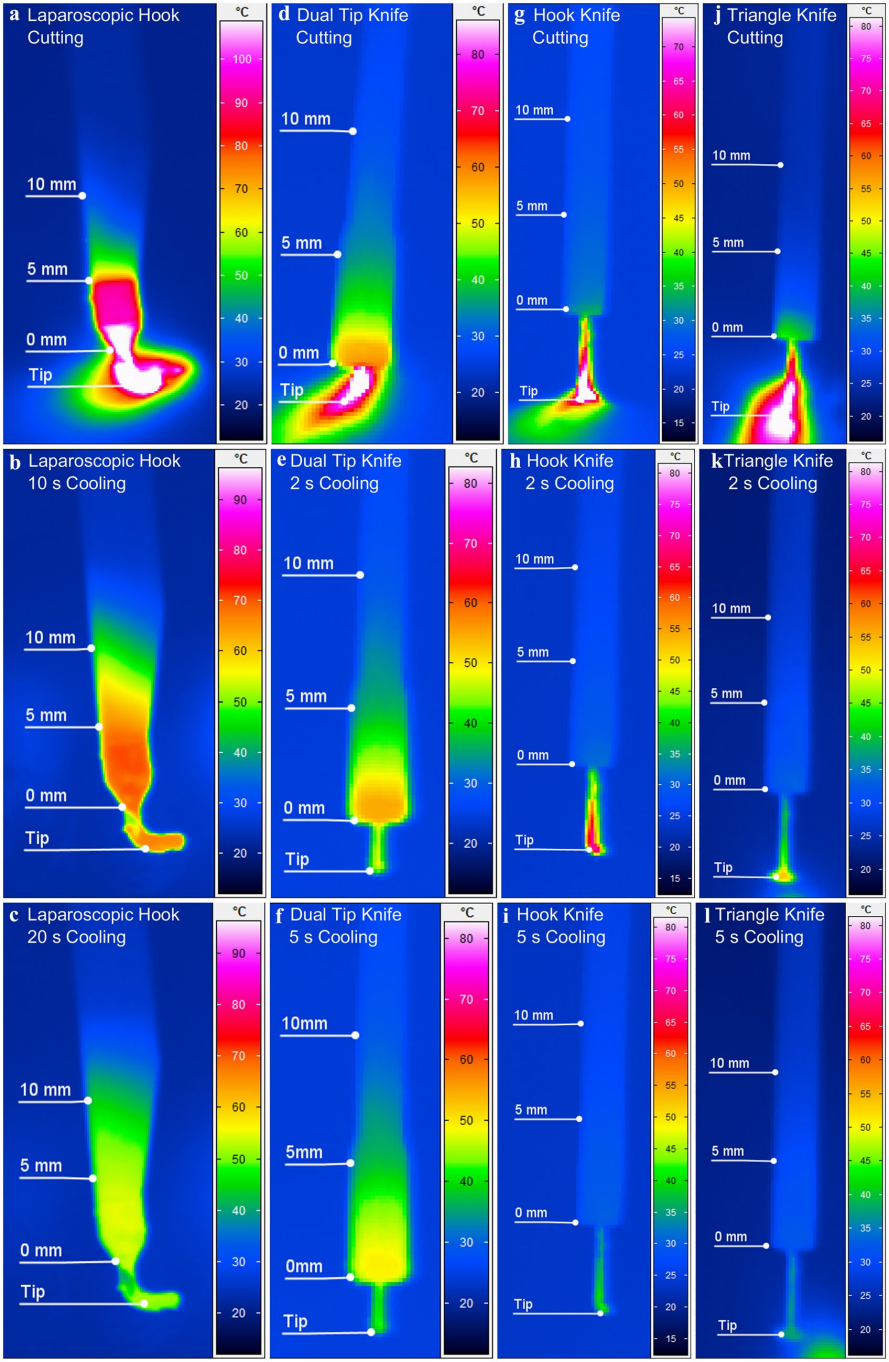
Thermogram of monoplar electrosurgical devices during and after energy application. Power Level: preciseSect effect 5 for Laparoscopy and EndocutQ effect 3 for Endoscopy. **a** Laparoscopic hook during energy application. **b** Laparoscopic hook after 10 s cooling. **c** Laparoscopic hook after 20 s cooling. **d** Dual Tip Knife during energy application. **e** Dual Tip Knife after 2 s cooling. **f** Dual Tip Knife after 2 s cooling. **g** Hook Knife during energy application. **h** Hook Knife after 2 s cooling. **i** Hook Knife after 5 s cooling. **j** Triangle Knife during energy application. **k** Triangle Knife after 2 s cooling. **l** Triangle Knife after 5 s cooling

To summarize and visualize our findings, we have compiled thermogram images of the instruments during energy application and in different cooling phases in Fig. 3.

### Maximum activation temperature of the instruments

Analyzing and considering the maximum activation temperature of the entire instrument is important to avoid tissue damage through accidental contact with the instrument’s shaft. Maximum temperatures during energy application classified by effect and cutting intervals are summarized in Table 1.

**Table 1:** Maximum activation temperature classified by effect and cutting interval

Instrument	Position	Maximum temperature in °C By effect Mean ± standard deviation			*p* value	Maximum temperature in °C By cutting cycle Mean ± standard deviation				*p* value
		Modus: PreciseSect laparoscopic dissection				Cutting duration in s				
		Effect 5	Effect 7	Effect 10		1 s	3 s	5 s	10 s	
L-Hook Laparoscopy	Tip	250 ± 15	254 ± 0	254 ± 0	NF	254 ± 0	248 ± 18	254 ± 0	254 ± 0	NF
	0 mm	121 ± 33	161 ± 20	180 ± 25	** < 0.001**	130 ± 35	146 ± 41	164 ± 16	177 ± 37	**0.036**
	5 mm	70 ± 15	101 ± 25	109 ± 19	** < 0.001**	87 ± 26	91 ± 35	86 ± 9	110 ± 28	0.224
	10 mm	41 ± 5	52 ± 8	56 ± 9	** < 0.001**	48 ± 10	50 ± 14	46 ± 3	54 ± 9	0.394

		Modus: EndoCutQ		*p* value	Cutting duration in s				*p* value
		Effect 3	Effect 4		1 s	3 s	5 s	10 s	
Triangle Tip Knife Endoscopy	Tip	229 ± 34	231 ± 28	0.26	216 ± 37	215 ± 21	235 ± 35	256 ± 0	0.094
	0 mm	45 ± 19	54 ± 16	**0.03**	49 ± 22	42 ± 9	51 ± 15	57 ± 24	0.617
	5 mm	31 ± 3	33 ± 4	0.10	32 ± 4	31 ± 3	33 ± 5	32 ± 5	0.818
	10 mm	28 ± 1	29 ± 2	0.56	28 ± 2	28 ± 1	29 ± 2	28 ± 1	0.823
Dual tip Knife Endoscopy	Tip	201 ± 47	246 ± 26	** < 0.001**	179 ± 46	226 ± 47	239 ± 26	248 ± 19	**0.018**
	0 mm	77 ± 15	88 ± 26	0.24	56 ± 6	76 ± 8	98 ± 20	100 ± 11	** < 0.001**
	5 mm	36 ± 3	38 ± 4	0.19	33 ± 1	37 ± 3	38 ± 4	41 ± 2	** < 0.001**
	10 mm	30 ± 2	31 ± 3	0.14	28 ± 1	28 ± 1	30 ± 1	33 ± 23	**0.002**
Hook tip Knife Endoscopy	Tip	225 ± 39	230 ± 27	0.72	190 ± 18	255 ± 0	227 ± 33	239 ± 26	** < 0.001**
	0 mm	45 ± 8	47 ± 10	0.52	38 ± 3	44 ± 9	49 ± 11	52 ± 6	**0.028**
	5 mm	30 ± 2	31 ± 4	0.22	28 ± 1	30 ± 1	33 ± 6	31 ± 1	**0.048**
	10 mm	27 ± 1	28 ± 2	0.12	27 ± 1	27 ± 1	29 ± 3	28 ± 1	**0.038**

Bold text highlights a *p* < 0.05

Statistical significance was calculated using the Mann–Whitney-*U*-test/Wilcoxon-test for two groups and two-way analysis of variance (ANOVA) for comparing means of more than two groups

*NF* not feasible due to camera restriction at 255 °C

#### Maximum temperature of the laparoscopic hook

**Fig. 4:**
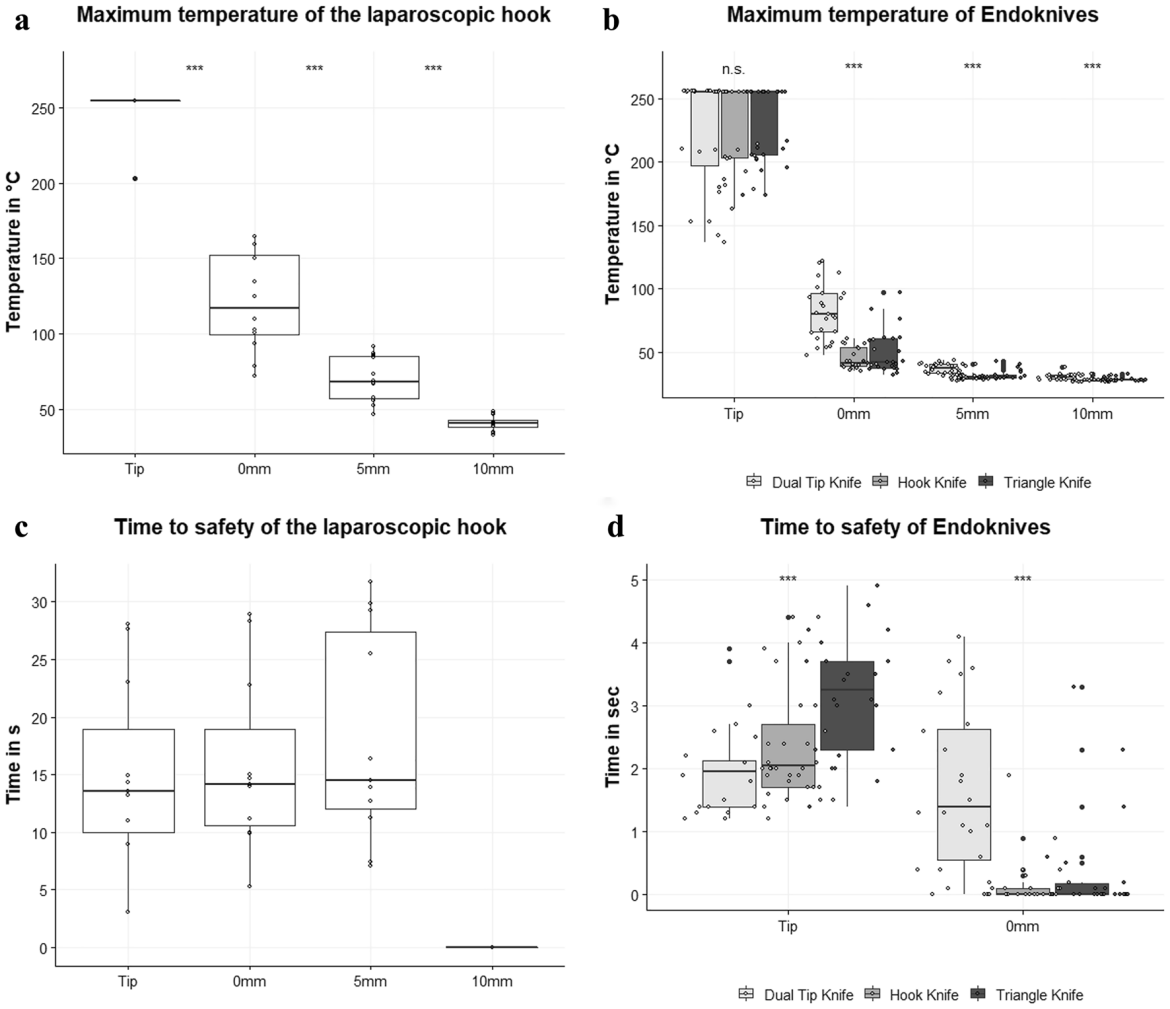
Maximum temperature and residual heat of monopolar instruments. **a** Maximum temperature during energy application of a monopolar laparoscopic hook. **b** Maximum temperature in-between the endoknives. **c** Time to safety defined as the time the instrument needed to cool down to below 50 °C after energy application of a laparoscopic hook. **d** Time to safety defined as the time the instrument needed to cool down to below 50 °C in-between the endoknives. ***:*p* < 0.001. *NA* not available as 50 °C have not been exceeded. *n.s.* not significant

In all performed cycles of laparoscopic dissection, maximum temperatures at the tip during energy application exceeded the detection limit of the infrared camera of 255 °C. The results for the most common clinical setting (preciseSect, effect 5) are shown in Fig. 4a. We observed that with the standard effect 5 the instrument already heats up to over 255 °C at the tip, to 121 ± 33 °C at 0 mm shaft length and to 70 ± 15 °C at 5 mm shaft length. At the 10 mm shaft length mark the temperature did not exceed 50 °C in effect 5. The maximum temperature of the shaft was significantly elevated with more powerful effects chosen (0 mm shaft length: effect 5: 121 ± 33 °C vs effect 7: 161 ± 20 °C vs. effect 10: 180 ± 25 °C, *p* < 0.001; 5 mm shaft length: effect 5: 70 ± 15 °C vs. effect 7: 101 ± 25 °C vs. effect 10: 109 ± 19 °C, *p* < 0.001). In all tested power levels, the maximum temperature regularly exceeded 50 °C at the tip, 0 mm shaft length as well as at 5 mm shaft length. With higher power levels the instrument heats up to over 50 °C at the 10 mm shaft length mark (10 mm shaft length, effect 5: 41 ± 5 °C vs. effect 7: 52 ± 8 °C vs effect 10: 56 ± 9 °C, *p* < 0.001). Higher maximum temperatures were recorded for longer cutting durations at the beginning of the shaft (0 mm shaft length, 1 s: 130 ± 35 °C vs. 3 s: 146 ± 41 °C vs. 5 s: 164 ± 16 °C vs. 10 s: 177 ± 37 °C, *p* = 0.036) but not at other measurement points (Table 1).

#### Maximum temperature of endoknives

We detected no significant difference between the maximum temperatures of the endoknives at the tip (tip: Dual Knife 223 ± 44 °C, Hook Knife 228 ± 33 °C, Triangle Knife 230 ± 30 °C, *p* = 0.817, Fig. 4b). A temperature difference at the beginning of the shaft was measured with the Dual Tip Knife (with shorter knife length), reaching almost twice the maximum temperature of the Hook and Triangle Knife (0 mm shaft length: Dual Knife 83 ± 22 °C vs. Hook Knife 46 ± 9 °C vs. Triangle Knife 50 ± 18 °C, *p* < 0.001, Fig. 4b). At 5 mm and 10 mm shaft length, the Dual Tip Knife showed a higher maximum temperature compared to the other two endoscopic knives (*p* > 0.001). However, the temperature here was less than 50 °C and thus not clinically relevant. Comparing the instruments’ temperatures using different effects, we demonstrated a significantly higher temperature at the tip of the Dual Tip Knife with a higher effect (effect 3: 201 ± 47 °C vs. effect 4 246 ± 26 °C, *p* < 0.001), whereas at 0 mm shaft length the Triangle Knife reached a higher temperature with a higher effect (effect 3: 45 ± 19 °C vs. effect 4: 54 ± 16 °C, *p* = 0.03). The Hook Knife did not show a maximum temperature difference with altered effects. The Dual Knife and Hook Knife exhibited a significantly higher temperature during longer cutting time at all points while no significant difference was found for the Triangle Knife (Table 1).

### Residual heat and cooling

As it is vital for the examiner to know how long the instrument needs to cool down sufficiently, we recorded the residual heat of laparoscopic and endoscopic instruments over time. The time until the temperature cooled down to < 50 °C was defined as “time to safety” and is shown in Table 2, classified by effect and cutting time.

**Table 2:** Residual heat: time to safety classified by effect and cutting interval

Instrument	Position	Time to safety (50 °C) in s By effect Mean ± standard deviation			*p* value	Time to safety (50 °C) in s By cutting cycle Mean ± standard deviation				*p* value
		Modus: preciseSect laparoscopic dissection				Cutting duration in s				
		Effect 5	Effect 7	Effect 10		1 s	3 s	5 s	10 s	
L-Hook Laparoscopy	Tip	15.2 ± 7.9	34.4 ± 5.5	35.8 ± 5.6	** < 0.001**	22.6 ± 11.5	26.4 ± 15.0	30.4 ± 11.6	31.5 ± 6.7	0.49
	0 mm	15.8 ± 7.6	34.3 ± 4.4	34.8 ± 5.0	** < 0.001**	23.1 ± 11.1	26.4 ± 14.4	30.1 ± 10.1	30.9 ± 5.6	0.52
	5 mm	17.0 ± 8.9	36.4 ± 6.1	36.7 ± 7.3	** < 0.001**	22.9 ± 12.8	30.7 ± 13.5	31.3 ± 12.6	34.1 ± 6.8	0.35
	10 mm	< 50 °C	33.8 ± 8.2	32.9 ± 8.2	0.85	33.1 ± 13.4	33.9 ± 5.1	32.2 ± 12.9	33.8 ± 8.1	0.99

		Modus: EndoCutQ		*p* value	Cutting duration in s				*p* value
		Effect 3	Effect 4		1 s	3 s	5 s	10 s	
Triangle Tip Knife Endoscopy	Tip	2.8 ± 1.1	3.5 ± 0.8	0.08	3.3 ± 0.3	2.4 ± 0.8	3.6 ± 1.0	3.4 ± 1.2	0.14
	0 mm	0.9 ± 0.8	1.7 ± 2.3	0.69	0.8 ± 0.8	< 50 °C	1.0 ± 1.2	1.7 ± 2.3	0.90
	5 mm	< 50 °C	< 50 °C	NA	< 50 °C	< 50 °C	< 50 °C	< 50 °C	NA
	10 mm	< 50 °C	< 50 °C	NA	< 50 °C	< 50 °C	< 50 °C	< 50 °C	NA
Dual Tip Knife Endoscopy	Tip	2.0 ± 0.9	1.9 ± 0.5	0.64	1.3 ± 0.1	1.9 ± 0.5	2.7 ± 0.8	2.4 ± 0.8	**0.024**
	0 mm	1.5 ± 1.2	2.0 ± 1.4	0.34	0.2 ± 0.2	1.3 ± 0.7	2.5 ± 1.2	2.7 ± 0.9	** < 0.001**
	5 mm	< 50 °C	< 50 °C	NA	< 50 °C	< 50 °C	< 50 °C	< 50 °C	NA
	10 mm	< 50 °C	< 50 °C	NA	< 50 °C	< 50 °C	< 50 °C	< 50 °C	NA
Hook Tip Knife Endoscopy	Tip	2.6 ± 1.0	2.1 ± 0.3	0.15	2.1 ± 0.6	2.1 ± 0.6	2.7 ± 1.2	2.6 ± 1.0	0.59
	0 mm	0.2 ± 0.2	0.3 ± 0.3	0.52	< 50 °C	0.6 ± 0.3	1.2 ± 0.5	1.0 ± 0.2	0.39
	5 mm	< 50 °C	< 50 °C	NA	< 50 °C	< 50 °C	< 50 °C	< 50 °C	NA
	10 mm	< 50 °C	< 50 °C	NA	< 50 °C	< 50 °C	< 50 °C	< 50 °C	NA

Bold text highlights a *p* < 0.05

Time to safety was defined as the time the instrument needed to cool down to < 50 °C after energy application. Statistical significance was calculated using the Mann–Whitney-*U*-test/Wilcoxon-test for two groups and the test for analysis of variance (ANOVA) for comparing means of more than two groups

*NA* not available as 50 °C have not been exceeded

#### Residual heat of the laparoscopic hook

The laparoscopic hook (preciseSect effect 5) required 15.1 ± 7.9 s at the tip and 15.8 ± 7.6 s at the beginning of the shaft to cool down to < 50 °C after activation. At 5 mm shaft length the time to safety was slightly longer with 17.0 ± 8.9 s (Fig. 4c) and 50 °C were never exceeded at the 10 mm shaft length mark with effect 5. The average “time to safety” for the more powerful effects 7 and 10 were significantly increased (Tip, effect 5: 15.2 ± 7.9 s vs. effect 7: 34.4 ± 5.5 s vs. effect 10: 35.8 ± 5.6 s, *p* < 0.001; 0 mm, 5 mm, 10 mm in Table 2). No significant difference was reported between the different cutting cycles (Table 2).

#### Residual heat of the endoknives

Endoknives displayed an entirely different residual heat behavior than laparoscopic hooks as they cooled down to < 50 °C within 1–4 s after cutting (Fig. 4d). Even with EndoCutQ effect 4, the temperature did not persist above 50 °C at any measuring point for more than 4 s. Also, the “time to safety” did not alter significantly between the different effects. Comparing endoscopic knives among each other, we observed that the Triangle Knife exerted the longest cooling time at the tip (Time to safety, Tip: Dual Tip Knife 1.9 ± 0.7 s vs. Hook Knife 2.4 ± 0.9 s vs. Triangle Knife 3.2 ± 1.0 s, *p* < 0.001). At the 0 mm shaft mark the Dual Tip Knife required significantly longer to cool down to below 50 °C than the endoscopic Hook Knife and Triangle Knife (Time to safety, 0 mm Shaft: Dual Tip Knife 1.8 ± 1.3 s vs. Hook Knife 0.3 ± 0.3 s vs. Triangle Knife 1.1 ± 1.1 s, *p* < 0.001). None of the endoscopic knives showed a temperature of more than 50 °C for the 5 mm and 10 mm shaft marks. The Dual Tip Knife showed a significantly higher cooling time with longer cutting time. At the tip and at 0 mm shaft length, a significantly increased time to safety was observed with longer cutting durations (Table 2, Tip: 1 s Cutting: 1.3 ± 0.1 s vs. 3 s Cutting: 1.9 ± 0.5 s vs. 5 s Cutting: 2.4 ± 0.8 vs. 10 s Cutting: 2.7 ± 0.8 s, *p* = 0.024; 0 mm Shaft: 1 s Cutting: 0.2 ± 0.2 s vs. 3 s Cutting: 1.3 ± 0.7 s vs. 5 s Cutting: 2.5 ± 1.2 vs. 10 s Cutting: 2.7 ± 0.9 s, *p* < 0.001).

## Discussion

Endoscopic and laparoscopic electrosurgical devices are indispensable instruments for modern medicine but can cause relevant adverse events such as tissue injuries and burns due to residual heat. In this study, we analyzed the temperature profile of monopolar laparoscopic and endoscopic instruments including their shafts in detail and demonstrated the temperature rise at the instrument’s shaft to be a crucial parameter that needs consideration, especially during laparoscopic surgery. We validated that the highest temperature of the electrosurgical instruments while applying energy occurs at the tip of the instrument and it is exceeding 255 °C in almost any activation pattern and effect. The temperature of the shaft of a laparoscopic hook even exceeds 120 °C after only one second of energy application. With higher effects, the temperature of the shaft increases significantly from 120 to over 180 °C, whilst the cutting time itself had a minor impact on the maximum temperature. The residual heat analysis of the laparoscopic hook revealed a cooling time of more than 15 s necessary at the tip and the beginning of the shaft’s instrument for an adequate cooldown to below 50 °C. The “time to safety” increases to more than 30 s at higher effects. Concerning the maximum temperature of the endoknives, the Dual Tip Knife with its short 2 mm electrode exceeded a temperature of 50 °C at the shaft. However, the endoscopic Hook and Triangle Knife with their longer 4 mm electrode hardly exhibited an increase in temperature at the shaft of above 50 °C, especially at lower effects. Regarding the residual heat, endoknives revealed a completely different behavior than the laparoscopic hook. The cooling time for a temperature below 50 °C at the tip of the instrument requires a maximum of 4 s.

Underestimating the residual heat of a laparoscopic instrument might cause serious complications such as burns of the adjacent tissue. Therefore, surgeons should be aware of their instrument’s temperature profile and hence when it is safe to re-use the instrument in the according manner desired. We are in line with previous studies, identifying the residual heat of laparoscopic devices as a relevant problem [23, 24]. Thermal injury through denaturation of intracellular proteins and destruction of cell membranes can occur when cells are heated to above 45–50 °C [21, 22, 27]. However, the extent of damage depends on both, the maximum temperature and time of exposure. We assume that an adverse injury can take place with instrument temperatures of 50 °C or higher, which as our data demonstrates can occur for 15 s or more after energy application in case of the laparoscopic hook. Our findings are in line with a study by Govekar et al. that identified a relevant increase in tissue temperature after 10 s for monopolar dissection instruments [23]. Still, the tissue damage should be further examined as the temperature of the device decreases rapidly on contact due to the tissue’s moist surface. In this study, we tested the laparoscopic hook as the most common laparoscopic instrument. Other laparoscopic tools often have significant larger tips, e.g., the spatula electrode or the laparoscopic shears. The thermal capacity of those tools is much higher due to the larger volume of the tip as compared to the hook electrode. Thus, the larger electrodes will not heat up as much as smaller electrodes, but nevertheless, they need an equal or even longer cooling time to reduce the stored heat energy because of the higher thermal capacity.

Up to date, there is no detailed study of the residual heat of endoscopic dissection knives despite their potential risk to cause serious injuries [15–18]. Endoknives exhibited an elevated temperature above 50 °C for 1–4 s after energy application. The Triangle Knife has a higher heat capacity due to its larger electrode volume and therefore needs longer cooling times at the tip as compared to the other endoknives. The Dual Tip Knife shows a stronger heat rise at the shaft due to its short electrode (2 mm) and thus requires longer cooling times. In conclusion, touching adjacent tissue with endoknives should be avoided within the first seconds after cutting.

Another major complication from electrosurgery are thermal injuries to the directly adjacent tissue [9]. The highest temperature of the electrosurgical instrument occurs at the tip of the instrument and exceeds 255 °C in almost any activation pattern and effect. Hence, the tip should be handled with great care to avoid serious tissue damage [23, 25]. However, up to now, temperature profiles of the instrument shaft were lacking. We observed that a laparoscopic hook reaches a critical temperature over 120 °C at the shaft to up to the 10 mm mark and thus can potentially cause unintentional tissue damages. The endoscopic Triangle Knife and Hook Knife did not increase their temperature at the shaft, probably due to the longer electrode of 4 mm, and therefore are safe for tissue contact with the shaft while energy is applied. In contrast, the Dual Tip Knife with its electrode of only 2 mm in length reached an elevated temperature of over 50 °C at the beginning of the shaft. Cutting in confined spaces (e.g. in an endoscopic submucosa dissection) can lead to frequent contact between the shaft and surrounding tissue and might trigger burns [15, 18]. Therefore, physicians must carefully move the shaft of their laparoscopic and endoscopic (especially Dual Tip Knife) instruments during cutting, even in narrow restricted surgical areas and during brief cuts, in order to avoid unwanted tissue burns. With higher effects, even more intensive caution must be exercised to spare surrounding tissue with the instrument’s shaft and prevent severe burns concordant to previous studies [25]. Interestingly, our investigation showed only a slight increase in the temperature of the instrument at different activation times. This finding should be investigated in greater detail to assess the true impact of the cutting duration.

Despite its high clinical relevance, our study has three main limitations. First, all experiments were performed with porcine tissue at room temperature. The temperature of the tissue was 10–15 °C cooler than the body temperature, so that overall lower temperatures and faster cooling can be assumed. In real life scenarios even longer cooling periods must be considered. Further, we used an infrared camera to record the temperature profiles of the instruments. However, we did not measure the temperature within the tissue and therefore we cannot draw final conclusions about tissue damage, but can assume that an increased instrument temperature > 50 °C will also cause tissue damage, as suggested by comparable studies [27]. Moreover, only one electrosurgical method was tested with the monopolar dissection instruments. Future studies would need to cover a broader methodological spectrum and investigate temperature profiles of laparoscopes with other tips (e.g. spatula electrode or the ballpoint electrode), ultrasonic or bipolar instruments and other endoscopic instruments such as polypectomy snares, radiofrequency ablation and argon plasma coagulation.

In conclusion, laparoscopic dissection instruments have a higher residual heat and thus require a longer “time to safety” (≥ 15 s) than endoscopic dissection knives (1–4 s). Furthermore, the temperature of a laparoscopic hook rises to over 120 °C at the shaft during energy application and therefore its contact with surrounding tissue should be avoided. The temperature of the endoscopic dissection knives’ shaft increases to a clinically relevant extent only with the Dual Tip Knife. The chosen power level and the duration of activation significantly increased the shaft’s temperature and the residual heat, and consequently the cooling time. Our findings are highly relevant to clinical practice and should be considered while handling monopolar laparoscopic and endoscopic dissection instruments to further improve patient safety. In the future, these temperature profiles could help to design optimized endoscopes and laparoscopic tools.
